# A coordinated EU approach to informed access decisions: CAVOD process proposals – the possibility to turn concept into reality?

**DOI:** 10.1186/1750-1172-7-S2-A25

**Published:** 2012-11-22

**Authors:** Wills Hughes-Wilson

**Affiliations:** 1 SOBI – Swedish Orphan Biovitrum, EU Committee of Experts on Rare Diseases (EUCERD

## Background

The European orphan legislative framework has been successful in delivering approved new treatments since its establishment in 2000. But the data needed to reach a positive risk-benefit analysis for regulatory approval often falls short of that needed by Member States to justify reimbursed availability to patients. This gap leads to differences in availability of new orphan medicines. The CAVOD proposals build on more than 12 years of formal collaborative approaches to European orphan drug policy, which have consistently agreed on the need to gather and share information at a European level. They would establish a process for the exchange of knowledge from the earliest stage in a drug’s development, through to in-life outcomes after a treatment is available to patients, with the objective of bundling fragmented know-how to allow the timely production of well-informed decisions on national pricing and reimbursement, while respecting existing roles, responsibilities and competences.

## Methodology and content

The approach intends to optimise processes, notably at four key time points. All activities should be based on existing and planned roles, responsibilities and legislative frameworks, and in collaboration between all parties involved at Member State and EU level, including regulators, HTA bodies, payers, patients and the sponsor. Early dialogue between the EMA, regulators and HTA bodies should be instigated at the time of orphan designation and the assumption of significant benefit. This should continue through protocol assistance to the Marketing Authorisation and confirmation of the significant benefit. Between the CHMP opinion and the granting of the Marketing Authorisation, the available information on a drug should be gathered in a useable form and additional information requirements defined, together with a plan for development to be undertaken by the Marketing Authorisation Holder. This will give HTA bodies access to the widest pool of in-use data on a pan-European basis, although the appropriate methodological tools to evaluate orphan drugs will need to be developed.

## Proposed results

It is intended that the collaboration at European level on the clinical added value of an orphan medicinal product will bridge the gap of data generation and availability between what is needed to reach a positive risk-benefit analysis and what is needed to facilitate understanding of the appropriate positioning of a product in the therapeutic arsenal for a given rare condition within national healthcare systems. This should be a voluntary process carried out on a case-by-case basis. Uptake will be a key measure of success, because use of the system will largely depend on its ability to deliver a more streamlined approach. Implementation could start already where the elements are in place, while additional required elements are being developed.

